# Rare Encounter of Cerebral Fat Embolism Post Motor Vehicle Accident: A Case Report

**DOI:** 10.7759/cureus.41877

**Published:** 2023-07-14

**Authors:** Deepak Singh Peram Singh, Lim Wei Yuan

**Affiliations:** 1 Orthopaedics and Traumatology, Hospital Kajang, Kajang, MYS

**Keywords:** neurological dysfunction, supportive therapy, magnetic resonance imaging (mri), cerebral manifestation, macroglobules

## Abstract

Fat embolism syndrome (FES) is known to occur when fat macroglobules get embolized into the blood circulation, whereby they then get dispersed out to multiple organs including the brain. It’s typically diagnosed when the patient sustains a neurological dysfunction, respiratory insufficiency, and petechial rash, the classical triad of FES. Cerebral fat embolism occurrence is rarely seen. In this case report, a 20-year-old male was admitted due to a closed left midshaft femur fracture from a motor vehicle accident, and sustained cerebral manifestation of fat embolism syndrome 32 hours post the incident. It was noted that the patient had the classical triad of FES and intubation was done for airway protection. MRI revealed features of cerebral fat embolism. Interlocking nail fixation of the left femur was done for this patient on day three after admission. On day 15 post trauma, the patient was successfully extubated. Adequate supportive management was given and the patient's prognosis improved. As a practitioner, it is important to recognize and diagnose cerebral fat embolism as early as possible so as to have a much better outcome and to avoid any unnecessary investigation.

## Introduction

Fat embolism occurs when fat macroglobules enter the blood circulation. Fat embolism may result in fat embolism syndrome (FES) leading to multisystem dysfunction with an overall mortality of 5-15% [1]. It is rare for fat embolism to develop into FES involving the cerebral region in long bone fractures, and this has an incidence of 0.9-2% [2]. The classical triad of FES consists of respiratory insufficiency/distress, altered mental status, and petechial skin rash. The term cerebral fat embolism (CFE) is used to imply FES affecting the brain.

## Case presentation

**Figure 1:**
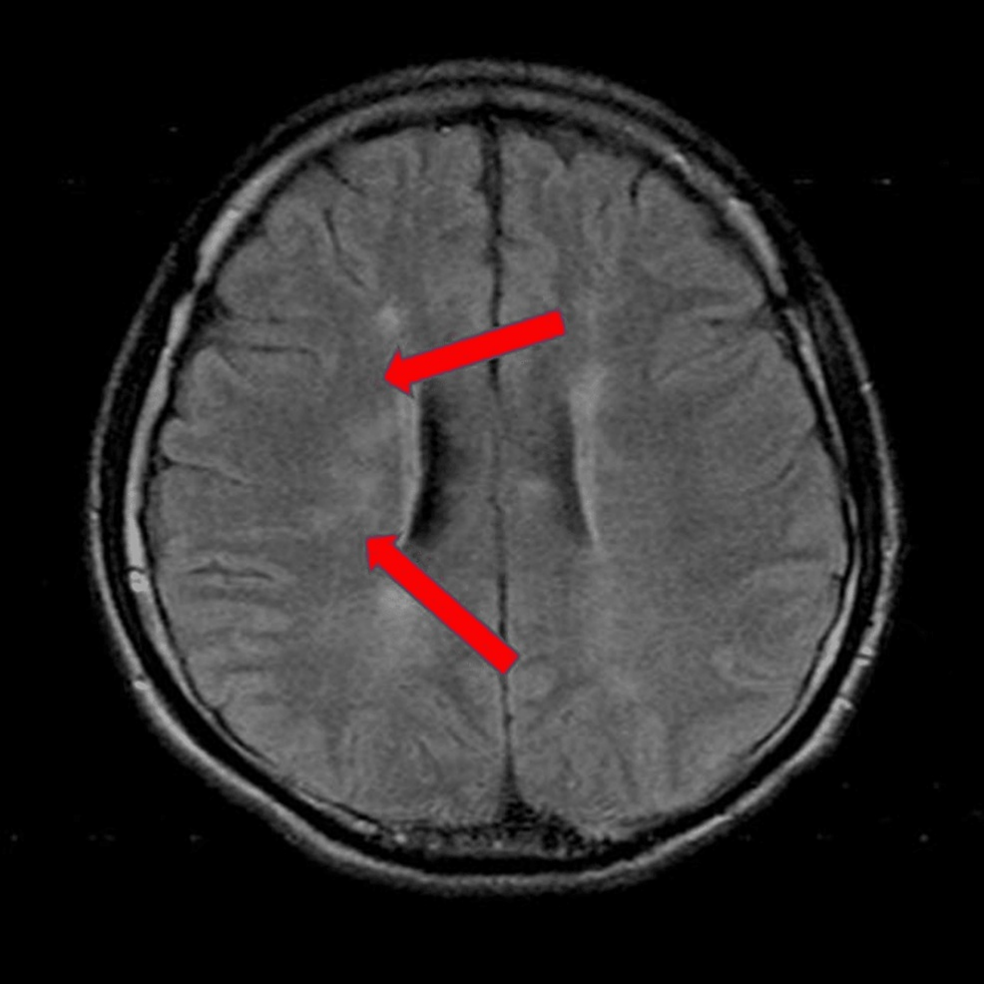
FLAIR image brain MRI scan showing patchy ill-defined subcentimeter hyperintensities scattered throughout bilateral frontoparietal lobe, predominantly at subcortical, periventricular, and deep white matter region FLAIR: fluid-attenuated inversion recovery

**Figure 2:**
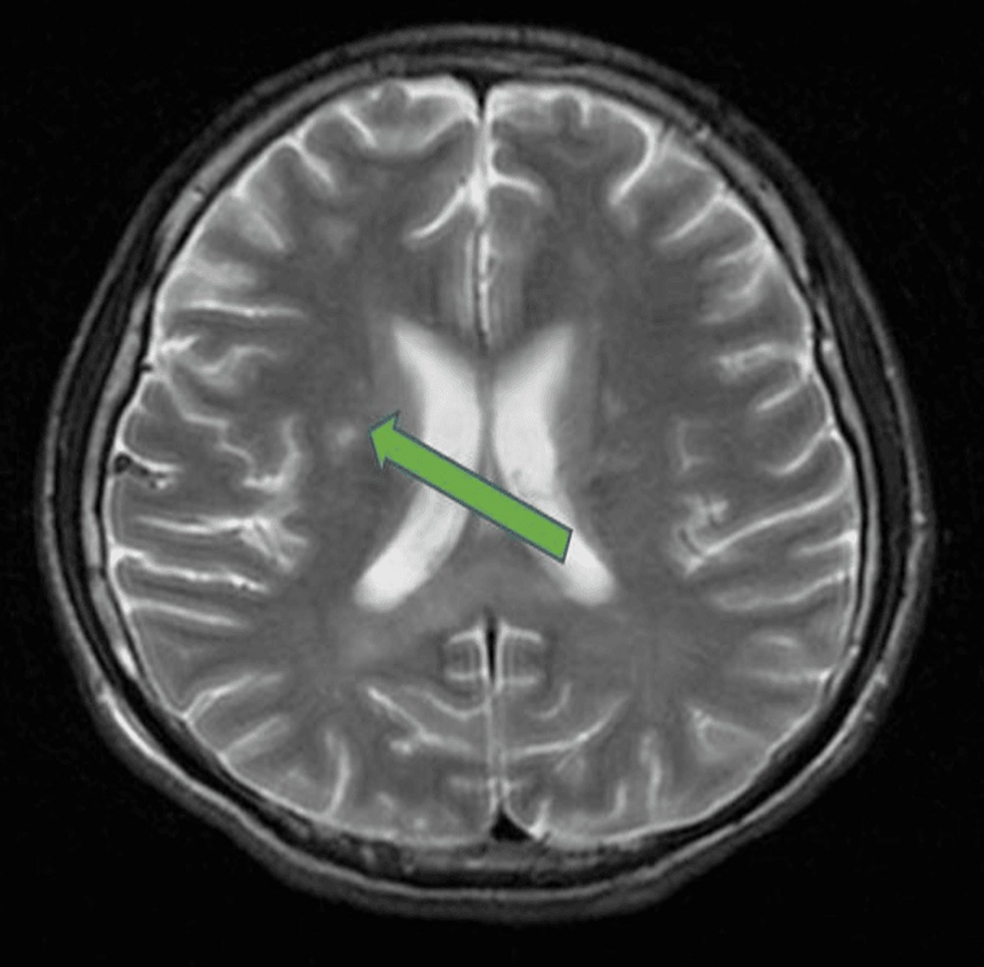
T2 weighted image brain MRI showing patchy ill-defined hyperintensities throughout the frontoparietal lobes, involving the subcortical, periventricular and deep white matter region

A 20-year-old male presented to the emergency department after a motor vehicle accident sustaining a close midshaft left femur fracture with multiple abrasion wounds. The patient also had a loss of consciousness during the accident but regained consciousness while on the way to the hospital. The GCS upon arrival to the hospital was E4V5M6 and the patient was able to open his eyes spontaneously, orientated to time, place, and person, and obey commands given. CT brain was done without administration of IV contrast and no abnormal pathology was noted. The patient was later put on skin traction and an IV drip was given. Thirty-two hours post trauma, the patient had confusion and become less responsive to commands. Vital signs included tachycardia with a heart rate of 110/minute and tachypnea with a respiratory rate of 26 breath/minute. Blood pressure was measured to be 107/72 mmHg and oxygen saturation was 90%. Petechial rashes were noted over the conjunctiva and thoracic region. GCS dropped further to E2V1M3 with eyes opening in response to pain, there was no verbal response, and abnormal flexion of his upper and lower limbs was distinguished. The patient was subsequently intubated for airway protection and admitted to intensive care unit (ICU) for the continuation of care. Urgent CT brain was done and no abnormal pathology was detected. Brain MRI was done on the same day and encountered an abnormal pathology finding on fluid-attenuated inversion recovery (FLAIR) (Figure 1) and T2 weighted (Figure 2).

Brain MRI scan showed patchy ill-defined subcentimeter hyperintensities scattered throughout the bilateral frontoparietal lobes, predominantly at subcortical, periventricular, and deep white matter regions. Differential diagnosis of diffuse axonal injury was considered but was ruled out as diffuse axonal injury usually affects the gray-white matter interface of the frontotemporal lobes and corpus callosum region. Based on the MRI finding and the fulfilment of the Gurd's clinical criteria, the patient was treated for CFE. Operative intervention was carried out on day three of admission with interlocking nail fixation of his left femur fracture. The patient was successfully extubated 15 days post operation with stable oxygen saturation. He had a drastic positive change in his behavior and personality. He was attentive, talkative, and cheerful. Oxygen therapy was finally stopped and he was eventually discharged five days post extubation with outpatient rehabilitation in the physiotherapy unit. At the post-discharge one-month follow-up in the orthopaedic clinic, complete neurological recovery was noted.

## Discussion

Fat embolism is usually asymptomatic. In symptomatic cases, the symptoms usually manifest 24 to 72 hours after the injury occurs. Only in rare cases do the symptoms occur by 12 hours or two weeks later. The classical triad of FES is respiratory insufficiency, neurological impairment, and petechial rash. The incidence of symptomatic fat embolism has been reported in 1-29% of patients [1]. Death associated with FES has been reported in more than 80% [3]

FES is known to have a potentially lethal effect and varies in severity, whereby the outcomes range from complete recovery to death. The pathogenesis is still controversial with several theories proposed. It is stated that it occurs after a fat embolus passes through the pulmonary vasculature, which causes a systemic emboli formation that might affect the brain and the kidneys. The mechanical theory suggests that it is due to a large fat embolus that enters to the circulation system and thus causes the obstruction of the pulmonary vasculature system [4]. This is because fat cells have pro-thrombotic and pro-inflammatory properties, which cause rapid aggregation of platelets and increased fibrin generation, which end up causing a meshwork that gets lodged into the pulmonary artery circulation. The biochemical theory, on the other hand, states that hormonal changes after extensive trauma induce the hydrolysis of triglycerides and the release of free fatty acids, causing toxic endothelium damage in pulmonary capillary beds [5]. This would then lead to stimulation of the proinflammatory cytokine cascade, which would in turn lead to complications such as acute lung injury or acute respiratory distress syndrome.

The involvement of CFE is rare; however, there have been several reported cases. Cerebral manifestation due to FES is not specific and can be variable based on the individual and also the amount of embolus that gets lodged into the intracranial circulation. CFE usually occurs after fat embolus had penetrated into the arterial circulation, which can be explained by two theories. Firstly, it can happen when the fat globules enter into the arterial circulation from the right atrium into the left atrium directly through a shunt in the heart, which is through the patent foramen ovale (PFO) or arterial septal defect (ASD). This is known to be a paradoxical embolism occurrence [6]. Another way these fat globules can get into the arterial system is in the form of microglobules that would be filtered directly through the lung capillaries [4]. These microemboli are small and malleable (size <7-20 um), and thus would not cause any significant injury in the pulmonary aspect, but to the brain structure. Neurological dysfunction following the occurrence of CFE varies significantly due to adipose embolization in the brain, which could range from confusion to encephalopathy with coma and seizures. CFE is usually a clinical diagnosis, but specific findings on neuroimaging procedures may be a good tool in coming to a diagnosis of CFE. Differential diagnoses which may appear to have similar signs and symptoms to CFE such as blunt carotid injury, diffuse axonal injury, and also vertebrobasilar artery thrombosis should always be considered. Isolated neurological damage due to CFE is also possible, without any pulmonary involvement.

The gold standard tool in helping to establish the complication of CFE is MRI [7]. Studies have shown that CT is not so sensitive and specific enough. The characteristic finding on an MRI is the presence of numerous tiny punctate or confluent hyperintensities in the basal ganglia, periventricular white matter, and grey matter junction on the diffusion-weighted image. These signal abnormalities can be noticed in the both grey and white matter of the brain structure. These lesions usually disappear slowly within a few weeks to a few months.

The treatment of FES is usually supportive management, such as maintenance of adequate oxygenation, proper hydration, adequate hemodynamic stability, and prevention of long-term complications such as DVT and gastric ulcer. Management of FES is mainly supportive but there are cases reported successfully treated with intravenous steroids (methylprednisolone) [8] and mechanical thrombectomy for CFE [9]. If the patient suffered from CFE, complete neurological recovery is usually possible after about two to three months from the initial insult [10]. Studies conducted around the world also showed that neuropsychological evaluation was normal with patients who had CFE except for some who had borderline frontal dysfunction occurrence [10].

## Conclusions

Medical practitioners must always keep in mind that establishing the diagnosis of CFE early would have a much better outcome with complete recovery, but can also be fatal if it was not diagnosed during the early stages. In addition, with appropriate treatment and supportive therapy, recovery from FES is achievable. It is also important to identify FES early to prevent any unnecessary investigation being done.

## References

[1] Shaikh N (2009). Emergency management of fat embolism syndrome. J Emerg Trauma Shock.

[2] Bulger EM, Smith DG, Maier RV, Jurkovich GJ (1997). Fat embolism syndrome. A 10-year review. Arch Surg.

[3] Lee YS, Park SH, Hamm IS (2004). Cerebral fat embolism with multiple rib and thoracic spinal fractures. J Korean Neurosurg Soc.

[4] Gauss H (1924). The pathology of fat embolism. Arch Surg.

[5] Lehman EP, Moore RM (1927). Fat embolism: including experimental production without trauma. Arch Surg.

[6] Piuzzi NS, Zanotti G, Comba FM, Buttaro MA, Piccaluga F (2014). Paradoxical cerebral fat embolism in revision hip surgery. Case Rep Orthop.

[7] Rutman AM, Rapp EJ, Hippe DS, Vu B, Mossa-Basha M (2017). T2*-Weighted and diffusion magnetic resonance imaging differentiation of cerebral fat embolism from diffuse axonal injury. J Comput Assist Tomogr.

[8] Shariff MS, Ngadiron H, Hayati F, Ariffin AC (2017). Fat embolism syndrome treated with methylprednisolone: A different perception or a misconception. Borneo J Med Sci.

[9] Maïer B, Badat N, Boulouis G, Zuber M (2019). Mechanical thrombectomy for a cerebral fat embolism. Intensive Care Med.

[10] Dunkel J, Roth C, Erbguth F, Dietrich W, Hügens-Penzel M, Ferbert A (2017). Cerebral fat embolism: clinical presentation, diagnostic steps and long-term follow-up. Eur Neurol.

